# Draft Genome Sequence of *Endocarpon pusillum* Strain KoLRILF000583

**DOI:** 10.1128/genomeA.00452-14

**Published:** 2014-05-22

**Authors:** Sook-Young Park, Jaeyoung Choi, Gir-Won Lee, Chan-Ho Park, Jung A Kim, Soon-Ok Oh, Yong-Hwan Lee, Jae-Seoun Hur

**Affiliations:** a Korean Lichen Research Institute, Sunchon National University, Suncheon, South Korea; b Center for Fungal Pathogenesis, Fungal Bioinformatics Laboratory, Department of Agricultural Biotechnology, Seoul National University, Seoul, South Korea; c Research Institute for Agriculture and Life Sciences, Seoul National University, Seoul, South Korea; d Center for Fungal Genetic Resources and Plant Genomics and Breeding Institute, Seoul National University, Seoul, South Korea

## Abstract

Lichen *Endocarpon pusillum* is a highly desiccation-tolerant and dominant species in biological soil crusts in arid and semi-arid regions. We report the draft genome sequence of a lichen-forming fungus, *E. pusillum* strain KoLRILF000583. The draft genome assembly has a size of 37,173,200 bp with a GC content of 49.71%, consisting of 40 scaffolds.

## GENOME ANNOUNCEMENT

Biological soil crusts (BSCs) are ubiquitous microbial communities covering most terrestrial surfaces, especially in arid and semi-arid regions. In desert regions, the soil crust serves to restore desert ecosystems by stabilizing the soil against water and wind erosion (1, 2) and increasing carbon and nitrogen fixation (3). Indeed, the soil crusts effectuate the reduction of sand and dust storms and increase soil fertility (4–8). Lichens are common constituents of crust communities, alongside various other nonlichen fungi, bacteria, bryophytes, and algae. Lichens are composite organisms formed by symbiotic associations between a fungus (mycobiont) and an alga or a cyanobacterium (photobiont). As lichens are self-sufficient, autotrophic organisms, lichens are good candidates for the initial colonization and restoration of sandy soils in desert regions.

The lichen *Endocarpon pusillum* is a dominant species among the BSC community in desert regions (9). A number of studies have been conducted where *E. pusillum* was allowed to colonize sandy soils to prevent sand and dust storms and to increase soil fertility (9, 10), because *E. pusillum* exhibits stronger desiccation tolerance than other lichens. However, the information currently available is insufficient to understand the desiccation tolerance of *E. pusillum* at the molecular level. Although a strain of *E. pusillum* has been sequenced and analyzed previously (11), sequencing the genomes of several isolates may help us to understand the evolution of desiccation tolerance within this species.

The strain *E. pusillum* KoLRILF000583 was isolated from soil in Cuiliugou county (N37°25′49.6″ N104°34′59.5″), Ningxia Province, China, in 2010. DNA from axenic culture of the fungus was extracted using a DNeasy minikit (Qiagen, Valencia, CA). Draft sequencing was performed by the Illumina HiSeq 2000 system using a whole-genome shotgun strategy (Macrogen, Inc., Seoul, South Korea). The total length of the assembled genome of *E. pusillum* strain KoLRIF000583 was 37,173,200 bp, with a GC content of 49.71%, representing a 556-fold coverage. The genome was assembled into 469 contigs using Allpaths-LG assembler (version 47503) (12). The generated contigs were assembled into 40 scaffolds (≥1,000 bp) by using SPPACE (version 2.0) (13). Subsequent gene prediction analysis using MAKER (14) yielded a total of 12,062 protein-coding genes. Using the previously developed three gene family pipelines (15–17), 790 transcription factor (TF) genes, 91 cytochrome P450 genes, and 2,031 genes encoding secretory proteins were predicted. In addition, 15 putative polyketide synthase genes, containing ketoacyl synthase, acyltransferase, and acyl carrier domains, were predicted by domain search (18). The genome sequence of *E. pusillum* strain KoLRILF000583 would be a valuable resource for identifying desiccation-tolerance-related genes. Furthermore, the genome sequence will also serve as a platform to facilitate comparative genomics with other lichen-forming fungi as well as other species in the phylum *Ascomycota*.

### Nucleotide sequence accession numbers.

The draft genome sequence of *E. pusillum* KoLRILF000583 has been deposited at GenBank under accession number JFDM00000000. The version described in this article is the first version, JFDM01000000. The scaffold sequences were also deposited in GenBank under accession numbers KK106941 to KK106980 (40 scaffolds).
